# Diaqua­bis­{3-[4-(1*H*-imidazol-1-yl)phenyl]-5-(pyridin-2-yl-κ*N*)-1*H*-1,2,4-triazol-1-ido-κ*N* ^1^}zinc

**DOI:** 10.1107/S1600536812035428

**Published:** 2012-08-23

**Authors:** You-Song Wang, Guang-Mei Qiu, Cui-Juan Wang

**Affiliations:** a Department of Chemistry and Chemical Engineering, College of Life Science and Bioengineering, SouthWest JiaoTong University, Chengdu, Sichuan 610031, People’s Republic of China

## Abstract

The centrosymmetric mol­ecule of the title compound, [Zn(C_16_H_11_N_6_)_2_(H_2_O)_2_], contains one Zn^2+^ ion located on a center of symmetry, two 3-[4-(1*H*-imidazol-1-yl)phen­yl]-5-(pyridin-2-yl)-1*H*-1,2,4-triazol-1-ide (Ippyt) ligands and two coordinating water mol­ecules. The Zn^II^ ion is six-coordinated in a distorted octa­hedral coordination geometry by four N atoms from two Ippyt ligands and by two O atoms from two water mol­ecules. Adjacent units are inter­connected though O—H⋯N hydrogen bonds, forming a three-dimensional network.

## Related literature

For similar structures, see: Braga *et al.* (2005 ▶); Lin *et al.* (2010 ▶); Faulmann *et al.* (1990 ▶); Han *et al.* (2005 ▶); Xue *et al.* (2009 ▶).
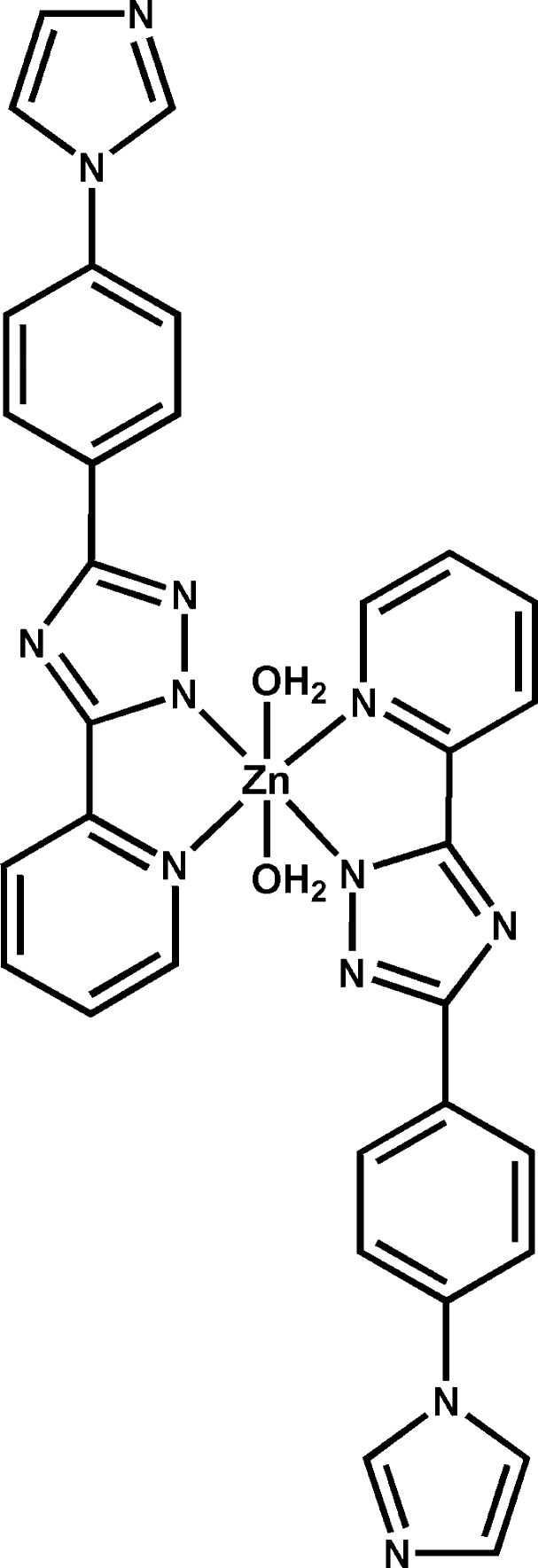


## Experimental

### 

#### Crystal data

[Zn(C_16_H_11_N_6_)_2_(H_2_O)_2_]
*M* *_r_* = 676.04
Monoclinic, 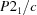

*a* = 12.6481 (9) Å
*b* = 11.6659 (6) Å
*c* = 10.4922 (7) Å
β = 105.891 (7)°
*V* = 1488.98 (16) Å^3^
*Z* = 2
Mo *K*α radiation
μ = 0.88 mm^−1^
*T* = 293 K
0.03 × 0.03 × 0.02 mm

#### Data collection

Bruker SMART diffractometer
Absorption correction: multi-scan (*SADABS*; Sheldrick, 1996 ▶) *T*_min_ = 0.974, *T*_max_ = 0.983
4938 measured reflections
2626 independent reflections
1724 reflections with *I* > 2σ(*I*)
*R*_int_ = 0.048

#### Refinement

*R*[*F* ^2^ > 2σ(*F* ^2^)] = 0.049
*wR*(*F* ^2^) = 0.109
*S* = 1.02
2626 reflections
214 parameters
H-atom parameters constrained
Δρ_max_ = 0.23 e Å^−3^
Δρ_min_ = −0.29 e Å^−3^

### 

Data collection: *SMART* (Bruker, 1997 ▶); cell refinement: *SAINT* (Bruker, 1997 ▶); data reduction: *SAINT*; program(s) used to solve structure: *SHELXS97* (Sheldrick, 2008 ▶); program(s) used to refine structure: *SHELXL97* (Sheldrick, 2008 ▶); molecular graphics: *DIAMOND* (Brandenburg, 2005 ▶); software used to prepare material for publication: *SHELXTL* (Sheldrick, 2008 ▶).

## Supplementary Material

Crystal structure: contains datablock(s) I, global. DOI: 10.1107/S1600536812035428/br2206sup1.cif

Structure factors: contains datablock(s) I. DOI: 10.1107/S1600536812035428/br2206Isup2.hkl

Additional supplementary materials:  crystallographic information; 3D view; checkCIF report

## Figures and Tables

**Table 1:** Hydrogen-bond geometry (Å, °)

*D*—H⋯*A*	*D*—H	H⋯*A*	*D*⋯*A*	*D*—H⋯*A*
O1—H1⋯N6^i^	0.82	2.03	2.842 (4)	174
O1—H1*B*⋯N4^ii^	0.85	2.07	2.868 (4)	157

Symmetry codes: (i) 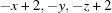; (ii) 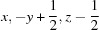.

**Fig. 1.**
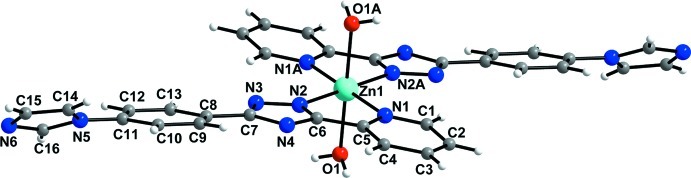
The coordination environment of ZnII atom in the title compound.

**Fig. 2.**
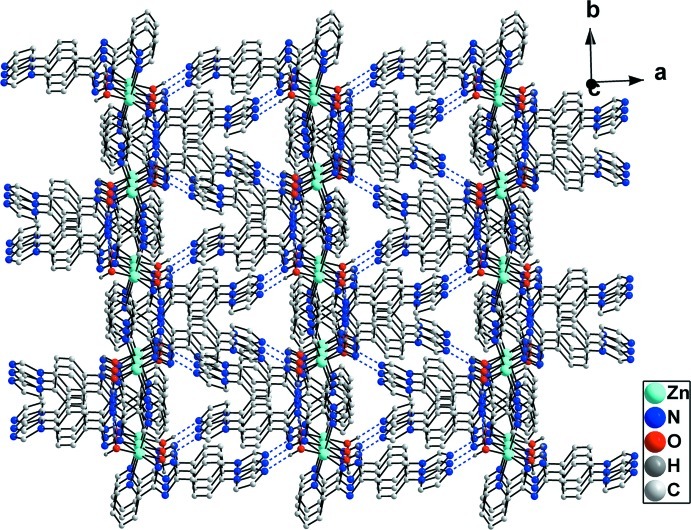
The 3D supermolecule network of the title compound. Dashed lines denote hydrogen bonds.

## supplementary crystallographic information

### Comment

The rational design and syntheses of metal-organic frameworks have been of increasing interest in the crystal engineering of coordination polymers owing to their ability to provide diverse assemblies with fascinating topological structures and material properties ( Han *et al.*, 2005; Xue *et al.*,2009). The centrosymmetric unit of the title compound contains one Zn^2+^ion, two Ippyt ligands and two coordination water molecules. For a similar structure, see: Braga *et al.* (2005); Lin *et al.* (2010); Faulmann *et al.* (1990). Every Zn^II^ ion is six-coordinated in a distorted octahedral coordination geometry by four N atoms from two Ippyt ligands and by two O atoms from two coordination water molecules (Fig. 1). There are two kinds of hydrogen bonding interactions which are between the coordinated waters and the triazolyl nitrogen atoms, and between the coordinated waters and the imidazolyl nitrogen atoms, respectively. However, the construct units are connected by the hydrogen bonding interactions between oxygen/ imidazolyl nitrogen atoms and imidazolyl nitrogen/ oxygen atoms from adjacent units respectively. Thus, infinite one-dimensional ring-shaped chains are formed. And then N3 and N3' are further involved in forming another hydrogen bonding interactions with other neighbouring water oxygen atoms and thus connect the 1D supramolecular chains together to form the two-dimensional supramolecular architecture in the a,c plane. And finally the structures are interlinked alternately by different hydrogen bonding interactions and finally result in the three-dimensional supramolecular network architectures.(Fig.2).

### Experimental

A mixture of Zn(NO_3_)_2_.6H_2_O (0.02 mmol), Ippyt (0.02 mmol), H2O (8 ml) was sealed in 25ml Teflon-lined stainless steel reactor, which was heated to 413 K for 5d and was subsequently cooled slowly to room temperature. Colourless block-shaped crystals were collected in 47% yield based on Zn.

### Refinement

All H atoms were positioned geometrically (C-H = 0.93Åand O-H = 0.82 Å) and allowed to ride on their parent atoms, with *U*_iso_(H) values equal to 1.2*U*_eq_(C) or 1.5U_eq_(O).

### Crystal data

**Table:**

[Zn(C_16_H_11_N_6_)_2_(H_2_O)_2_]	*F*(000) = 700
*M**_r_* = 676.04	*D*_x_ = 1.512 Mg m^−^^3^
Monoclinic, *P*2_1_/*c*	Mo *K*α radiation, λ = 0.71073 Å
Hall symbol: -P 2ybc	Cell parameters from 1141 reflections
*a* = 12.6481 (9) Å	θ = 2.4–28.3°
*b* = 11.6659 (6) Å	µ = 0.88 mm^−^^1^
*c* = 10.4922 (7) Å	*T* = 293 K
β = 105.891 (7)°	Block, colourless
*V* = 1488.98 (16) Å^3^	0.03 × 0.03 × 0.02 mm
*Z* = 2	

### Data collection

**Table:**

Bruker SMART diffractometer	2626 independent reflections
Radiation source: fine-focus sealed tube	1724 reflections with *I* > 2σ(*I*)
Graphite monochromator	*R*_int_ = 0.048
φ and ω scans	θ_max_ = 25.0°, θ_min_ = 2.4°
Absorption correction: multi-scan (*SADABS*; Sheldrick, 1996)	*h* = −15→8
*T*_min_ = 0.974, *T*_max_ = 0.983	*k* = −12→13
4938 measured reflections	*l* = −8→12

### Refinement

**Table:**

Refinement on *F*^2^	Secondary atom site location: difference Fourier map
Least-squares matrix: full	Hydrogen site location: inferred from neighbouring sites
*R*[*F*^2^ > 2σ(*F*^2^)] = 0.049	H-atom parameters constrained
*wR*(*F*^2^) = 0.109	*w* = 1/[σ^2^(*F*_o_^2^) + (0.0332*P*)^2^] where *P* = (*F*_o_^2^ + 2*F*_c_^2^)/3
*S* = 1.02	
2626 reflections	Δρ_max_ = 0.23 e Å^−^^3^
214 parameters	Δρ_min_ = −0.29 e Å^−^^3^
Primary atom site location: structure-invariant direct methods	

### Special details

**Table:**

Geometry. All esds (except the esd in the dihedral angle between two l.s. planes) are estimated using the full covariance matrix. The cell esds are taken into account individually in the estimation of esds in distances, angles and torsion angles; correlations between esds in cell parameters are only used when they are defined by crystal symmetry. An approximate (isotropic) treatment of cell esds is used for estimating esds involving l.s. planes.
Refinement. Refinement of F^2^ against ALL reflections. The weighted R-factor wR and goodness of fit S are based on F^2^, conventional R-factors R are based on F, with F set to zero for negative F^2^. The threshold expression of F^2^ > 2sigma(F^2^) is used only for calculating R-factors(gt) etc. and is not relevant to the choice of reflections for refinement. R-factors based on F^2^ are statistically about twice as large as those based on F, and R- factors based on ALL data will be even larger.

### Fractional atomic coordinates and isotropic or equivalent isotropic displacement parameters (Å^2^)

**Table:**

	*x*	*y*	*z*	*U*_iso_*/*U*_eq_	
Zn1	0.5000	0.0000	0.5000	0.0451 (2)	
N1	0.4454 (2)	0.1728 (2)	0.4816 (3)	0.0390 (8)	
N2	0.6022 (2)	0.0728 (2)	0.6718 (3)	0.0391 (8)	
N3	0.6852 (2)	0.0414 (3)	0.7797 (3)	0.0441 (8)	
N4	0.6357 (2)	0.2248 (2)	0.8079 (3)	0.0385 (8)	
N5	1.0281 (2)	0.1265 (3)	1.3567 (3)	0.0553 (9)	
N6	1.1782 (3)	0.0806 (3)	1.5144 (4)	0.0726 (12)	
O1	0.62155 (17)	0.04131 (19)	0.3861 (3)	0.0481 (7)	
H1B	0.6317	0.1134	0.3870	0.058*	
H1	0.6799	0.0087	0.4200	0.072*	
C1	0.3757 (3)	0.2195 (3)	0.3764 (4)	0.0469 (10)	
H1A	0.3432	0.1729	0.3045	0.056*	
C2	0.3496 (3)	0.3348 (3)	0.3698 (4)	0.0516 (11)	
H2	0.3024	0.3661	0.2938	0.062*	
C3	0.3952 (3)	0.4012 (3)	0.4776 (4)	0.0545 (11)	
H3	0.3769	0.4784	0.4767	0.065*	
C4	0.4682 (3)	0.3549 (3)	0.5884 (4)	0.0474 (10)	
H4	0.4994	0.4000	0.6623	0.057*	
C5	0.4938 (2)	0.2399 (3)	0.5865 (4)	0.0344 (8)	
C6	0.5757 (3)	0.1811 (3)	0.6919 (4)	0.0347 (8)	
C7	0.7019 (3)	0.1340 (3)	0.8583 (4)	0.0378 (9)	
C8	0.7859 (3)	0.1342 (3)	0.9879 (4)	0.0393 (9)	
C9	0.7952 (3)	0.2227 (3)	1.0774 (4)	0.0468 (10)	
H9	0.7472	0.2846	1.0566	0.056*	
C10	0.8756 (3)	0.2204 (3)	1.1987 (4)	0.0510 (11)	
H10	0.8815	0.2811	1.2578	0.061*	
C11	0.9465 (3)	0.1283 (3)	1.2313 (4)	0.0464 (10)	
C12	0.9361 (3)	0.0383 (4)	1.1451 (5)	0.0595 (12)	
H12	0.9823	−0.0249	1.1676	0.071*	
C13	0.8562 (3)	0.0414 (3)	1.0237 (4)	0.0559 (11)	
H13	0.8500	−0.0199	0.9655	0.067*	
C14	1.0221 (4)	0.1771 (6)	1.4698 (5)	0.113 (2)	
H14	0.9652	0.2232	1.4806	0.136*	
C15	1.1136 (4)	0.1485 (5)	1.5641 (5)	0.114 (2)	
H15	1.1299	0.1725	1.6519	0.137*	
C16	1.1249 (3)	0.0693 (4)	1.3884 (5)	0.0675 (13)	
H16	1.1507	0.0269	1.3280	0.081*	

### Atomic displacement parameters (Å^2^)

**Table:**

	*U*^11^	*U*^22^	*U*^33^	*U*^12^	*U*^13^	*U*^23^
Zn1	0.0514 (4)	0.0329 (3)	0.0373 (4)	0.0044 (3)	−0.0112 (3)	−0.0042 (4)
N1	0.0353 (16)	0.0368 (18)	0.037 (2)	0.0012 (14)	−0.0030 (14)	0.0003 (16)
N2	0.0400 (17)	0.0332 (17)	0.0352 (19)	0.0010 (13)	−0.0049 (14)	−0.0039 (15)
N3	0.0469 (18)	0.0380 (18)	0.036 (2)	0.0023 (14)	−0.0069 (15)	−0.0009 (17)
N4	0.0367 (16)	0.0354 (17)	0.0371 (19)	0.0015 (13)	−0.0005 (14)	−0.0029 (16)
N5	0.0367 (18)	0.082 (2)	0.040 (2)	0.0157 (17)	−0.0026 (15)	−0.002 (2)
N6	0.049 (2)	0.097 (3)	0.056 (3)	0.018 (2)	−0.0124 (19)	0.001 (3)
O1	0.0452 (14)	0.0400 (14)	0.0496 (18)	0.0068 (11)	−0.0032 (12)	0.0066 (14)
C1	0.048 (2)	0.048 (3)	0.033 (2)	0.0015 (18)	−0.0066 (18)	−0.001 (2)
C2	0.052 (2)	0.045 (2)	0.045 (3)	0.0087 (19)	−0.008 (2)	0.005 (2)
C3	0.059 (3)	0.038 (2)	0.056 (3)	0.0133 (19)	−0.002 (2)	0.000 (2)
C4	0.052 (2)	0.037 (2)	0.043 (3)	0.0054 (18)	−0.0035 (19)	−0.008 (2)
C5	0.0328 (19)	0.034 (2)	0.033 (2)	0.0002 (15)	0.0020 (16)	0.0004 (19)
C6	0.038 (2)	0.033 (2)	0.032 (2)	−0.0027 (16)	0.0065 (16)	−0.0031 (18)
C7	0.035 (2)	0.040 (2)	0.034 (2)	−0.0052 (16)	0.0028 (16)	−0.001 (2)
C8	0.034 (2)	0.042 (2)	0.036 (2)	0.0027 (16)	0.0006 (17)	0.002 (2)
C9	0.044 (2)	0.050 (2)	0.041 (3)	0.0092 (18)	0.0016 (18)	−0.003 (2)
C10	0.047 (2)	0.060 (3)	0.039 (2)	0.008 (2)	−0.0003 (19)	−0.015 (2)
C11	0.040 (2)	0.057 (3)	0.035 (2)	0.0064 (19)	−0.0021 (17)	0.001 (2)
C12	0.053 (3)	0.057 (3)	0.055 (3)	0.020 (2)	−0.007 (2)	−0.003 (3)
C13	0.056 (2)	0.050 (2)	0.051 (3)	0.009 (2)	−0.003 (2)	−0.010 (2)
C14	0.078 (4)	0.202 (7)	0.045 (3)	0.071 (4)	−0.008 (3)	−0.024 (4)
C15	0.074 (4)	0.209 (7)	0.041 (3)	0.057 (4)	−0.015 (3)	−0.018 (4)
C16	0.054 (3)	0.075 (3)	0.059 (3)	0.017 (2)	−0.009 (2)	−0.005 (3)

### Geometric parameters (Å, º)

**Table:**

Zn1—N2^i^	2.090 (3)	C2—C3	1.362 (5)
Zn1—N2	2.090 (3)	C2—H2	0.9300
Zn1—N1	2.123 (3)	C3—C4	1.381 (5)
Zn1—N1^i^	2.123 (3)	C3—H3	0.9300
Zn1—O1^i^	2.243 (2)	C4—C5	1.381 (4)
Zn1—O1	2.243 (2)	C4—H4	0.9300
N1—C1	1.326 (4)	C5—C6	1.463 (5)
N1—C5	1.353 (4)	C7—C8	1.479 (5)
N2—C6	1.339 (4)	C8—C9	1.378 (5)
N2—N3	1.366 (4)	C8—C13	1.385 (5)
N3—C7	1.340 (4)	C9—C10	1.396 (5)
N4—C6	1.346 (4)	C9—H9	0.9300
N4—C7	1.365 (4)	C10—C11	1.381 (5)
N5—C14	1.345 (6)	C10—H10	0.9300
N5—C16	1.353 (5)	C11—C12	1.368 (5)
N5—C11	1.433 (5)	C12—C13	1.393 (5)
N6—C16	1.316 (6)	C12—H12	0.9300
N6—C15	1.343 (6)	C13—H13	0.9300
O1—H1B	0.8500	C14—C15	1.342 (6)
O1—H1	0.8199	C14—H14	0.9300
C1—C2	1.382 (5)	C15—H15	0.9300
C1—H1A	0.9300	C16—H16	0.9300
			
N2^i^—Zn1—N2	180.0	C3—C4—C5	118.2 (4)
N2^i^—Zn1—N1	101.45 (11)	C3—C4—H4	120.9
N2—Zn1—N1	78.55 (11)	C5—C4—H4	120.9
N2^i^—Zn1—N1^i^	78.55 (11)	N1—C5—C4	121.2 (3)
N2—Zn1—N1^i^	101.45 (11)	N1—C5—C6	114.4 (3)
N1—Zn1—N1^i^	180.0	C4—C5—C6	124.4 (3)
N2^i^—Zn1—O1^i^	91.13 (10)	N2—C6—N4	113.4 (3)
N2—Zn1—O1^i^	88.87 (10)	N2—C6—C5	118.7 (3)
N1—Zn1—O1^i^	89.95 (10)	N4—C6—C5	127.8 (3)
N1^i^—Zn1—O1^i^	90.05 (10)	N3—C7—N4	114.3 (3)
N2^i^—Zn1—O1	88.87 (10)	N3—C7—C8	121.3 (3)
N2—Zn1—O1	91.13 (10)	N4—C7—C8	124.5 (3)
N1—Zn1—O1	90.05 (10)	C9—C8—C13	118.2 (3)
N1^i^—Zn1—O1	89.95 (10)	C9—C8—C7	122.2 (3)
O1^i^—Zn1—O1	180.0	C13—C8—C7	119.6 (4)
C1—N1—C5	119.3 (3)	C8—C9—C10	120.8 (3)
C1—N1—Zn1	126.3 (3)	C8—C9—H9	119.6
C5—N1—Zn1	114.3 (2)	C10—C9—H9	119.6
C6—N2—N3	107.0 (3)	C11—C10—C9	120.1 (4)
C6—N2—Zn1	113.4 (2)	C11—C10—H10	119.9
N3—N2—Zn1	139.4 (2)	C9—C10—H10	119.9
C7—N3—N2	104.4 (3)	C12—C11—C10	119.7 (4)
C6—N4—C7	101.0 (3)	C12—C11—N5	120.7 (3)
C14—N5—C16	105.4 (4)	C10—C11—N5	119.6 (4)
C14—N5—C11	127.1 (3)	C11—C12—C13	119.9 (4)
C16—N5—C11	127.4 (4)	C11—C12—H12	120.0
C16—N6—C15	104.5 (4)	C13—C12—H12	120.0
Zn1—O1—H1B	109.3	C8—C13—C12	121.3 (4)
Zn1—O1—H1	109.7	C8—C13—H13	119.4
H1B—O1—H1	109.8	C12—C13—H13	119.4
N1—C1—C2	122.5 (4)	C15—C14—N5	107.2 (4)
N1—C1—H1A	118.8	C15—C14—H14	126.4
C2—C1—H1A	118.8	N5—C14—H14	126.4
C3—C2—C1	118.1 (4)	C14—C15—N6	110.7 (5)
C3—C2—H2	121.0	C14—C15—H15	124.6
C1—C2—H2	121.0	N6—C15—H15	124.6
C2—C3—C4	120.7 (3)	N6—C16—N5	112.2 (4)
C2—C3—H3	119.7	N6—C16—H16	123.9
C4—C3—H3	119.7	N5—C16—H16	123.9

Symmetry code: (i) −*x*+1, −*y*, −*z*+1.

### Hydrogen-bond geometry (Å, º)

**Table:**

*D*—H···*A*	*D*—H	H···*A*	*D*···*A*	*D*—H···*A*
O1—H1···N6^ii^	0.82	2.03	2.842 (4)	174
O1—H1*B*···N4^iii^	0.85	2.07	2.868 (4)	157

Symmetry codes: (ii) −*x*+2, −*y*, −*z*+2; (iii) *x*, −*y*+1/2, *z*−1/2.
